# Quantification of Airborne Resistant Organisms With Temporal and Spatial Diversity in Bangladesh: Protocol for a Cross-Sectional Study

**DOI:** 10.2196/14574

**Published:** 2019-12-19

**Authors:** Muhammad Asaduzzaman, Muhammed Iqbal Hossain, Sumita Rani Saha, Md Rayhanul Islam, Niyaz Ahmed, Mohammad Aminul Islam

**Affiliations:** 1 Laboratory Sciences and Services Division International Centre for Diarrhoeal Disease Research, Bangladesh Dhaka Bangladesh; 2 School of Public Health University of California Berkeley, CA United States; 3 Centre for Global Health Institute of Health and Society University of Oslo Oslo Norway; 4 Pathogen Biology Laboratory, Department of Biotechnology and Bioinformatics School of Life Sciences University of Hyderabad Hyderabad India; 5 Paul G Allen School for Global Animal Health College of Veterinary Medicine Washington State University Pullman, WA United States

**Keywords:** antimicrobial resistance, airborne resistomes, air quality, global health, planetary health, environmental risk assessment

## Abstract

**Background:**

Antimicrobial resistance is a widespread, alarming issue in global health and a significant contributor to human death and illness, especially in low and middle-income countries like Bangladesh. Despite extensive work conducted in environmental settings, there is a scarcity of knowledge about the presence of resistant organisms in the air.

**Objective:**

The objective of this protocol is to quantify and characterize the airborne resistomes in Bangladesh, which will be a guide to identify high-risk environments for multidrug-resistant pathogens with their spatiotemporal diversity.

**Methods:**

This is a cross-sectional study with an environmental, systematic, and grid sampling strategy focused on collecting air samples from different outdoor environments during the dry and wet seasons. The four environmental compartments are the frequent human exposure sites in both urban and rural settings: urban residential areas (n=20), live bird markets (n=20), rural households (n=20), and poultry farms (n=20). We obtained air samples from 80 locations in two seasons by using an active microbial air sampler. From each location, five air samples were collected in different media to yield the total bacterial count of 3rd generation cephalosporin (3GC) resistant *Enterobacteriaceae*, carbapenem-resistant *Enterobacteriaceae*, vancomycin-resistant *Enterococci* and methicillin-resistant *Staphylococcus aureus*.

**Results:**

The study started in January 2018, and the collection of air samples was completed in November 2018. We have received 800 air samples from 80 study locations in both dry and wet seasons. Currently, the laboratory analysis is ongoing, and we expect to receive the preliminary results by October 2019. We will publish the complete result as soon as we clean and analyze the data and draft the manuscript.

**Conclusions:**

The existence of resistant bacteria in the air like those producing extended-spectrum beta-lactamases, carbapenem-resistant *Enterobacteriaceae*, vancomycin-resistant *Enterococci*, and methicillin-resistant *Staphylococcus aureus* will justify our hypothesis that the outdoor environment (air) in Bangladesh acts as a reservoir for bacteria that carry genes conferring resistance to antibiotics. To our knowledge, this is the first study to explore the presence of superbugs in the air in commonly exposed areas in Bangladesh.

**International Registered Report Identifier (IRRID):**

DERR1-10.2196/14574

## Introduction

Antimicrobial resistance is considered a rapidly progressive global public health issue with the potential of environmental transmission to a larger extent. However, very little information is available on the transmission of antimicrobial resistance through the air. Additionally, the capacity to carry and propagate resistance of these resistomes is poorly studied worldwide. There is a recognized need to examine the existence of such bacteria that have the ability to confer resistance through atmospheric air. Bangladesh is an important location to study this pathway. This study can provide critical insight into antimicrobial resistance transmission and help determine where efforts could be implemented to reduce environmental transmission.

Antimicrobial resistance is a widespread and alarming issue in global health, causing more than 700,000 deaths every year [1]. In Bangladesh, the insufficient and poor guidelines for the disposal of antibiotic residues into the environment from pharmaceuticals or clinical settings and the high population density have made its environment favorable for the wide dissemination of antimicrobial resistance. In addition to use in human therapy, antibiotics are used extensively in animal farming and eventually, a large amount of antibiotics and their residues are disseminated into the environment through both air and water [2]. Again, environmental contamination with human and animal-originated bacteria contributes significantly to the mechanism of development of extensive antimicrobial resistance [3]. This occurs when the unlimited genes contained in the bacteria circulating in air invade other pathogenic bacteria through mobile genetic elements and may be transformed to antibiotic resistance genes like integrons, transposons, and plasmids [4,5].

Based on few studies conducted on the presence of antimicrobial resistance in the environment, the spatial and seasonal diversity of antibiotic resistant bacteria are well established [6-11], but the diversity in air is not well studied. In case of detection of airborne resistomes, most of the studies have been conducted in clinical settings, pharmaceutical industries, animal farms, laboratory settings, or highly polluted sites [12-27]. However, there is a gap in the quantification of airborne resistomes in both high-risk and low-risk areas like urban versus rural areas and poultry/industry versus nonpoultry/residential areas in the same study area as well as their transmission dynamics with seasonal differences.

Additionally, several anthropogenic events also enhance the ability of antibiotic resistance genes to be transferred horizontally and pose a further risk for the environment to act as a reservoir for resistant bacteria [28,29]. Unfortunately, we are not quite aware of the presence of resistant genes in the air or their capability of transmission to humans. This needs to be explored, especially the transmission potential of pathogenic resistant bacteria [30]. Another less-exposed area of research is the climatic and seasonal variability (eg, humidity, rainfall, temperature) in the environmental transmission of antimicrobial resistance. Therefore, the quantity of organisms and diversity of antibiotic resistance genes will vary depending on the air in different environments and seasons. The alarming situation is the presence of the same genes in clinically ill patients, which is supported by different studies [31-33]. A study [34] conducted on the hospital air environment yielded 25% multidrug-resistant organisms. This study will address not only the presence of resistant organisms in the air, but also the clonal distribution of those organisms based on seasonal variation. Therefore, we will be able to identify the risky environments effectively.

The main study objective is to detect the existence of resistomes in the air samples from outdoor environments of Bangladesh, which carry genes that confer resistance to antibiotics with temporal and spatial diversity. We hypothesize that the outdoor environment (air) in Bangladesh acts as a reservoir for bacteria carrying genes that confer resistance to antibiotics with temporal and spatial diversity. The specific objectives of the study are as follows:

1. To determine the prevalence of antibiotic-resistant bacteria in the air samples of outdoor environments (both poultry and residential) in Bangladesh that carry genes conferring resistance to antibiotics.
2. To characterize antibiotic-resistant organisms using different phenotypic and genotypic methods.
3. To explore the clonal relationship between antimicrobial resistant organisms isolated from high- and low-risk areas.
4. To identify the temporal and spatial distribution of resistant bacteria in outdoor environments of Bangladesh.

## Methods

### Overview

**Figure 1:**
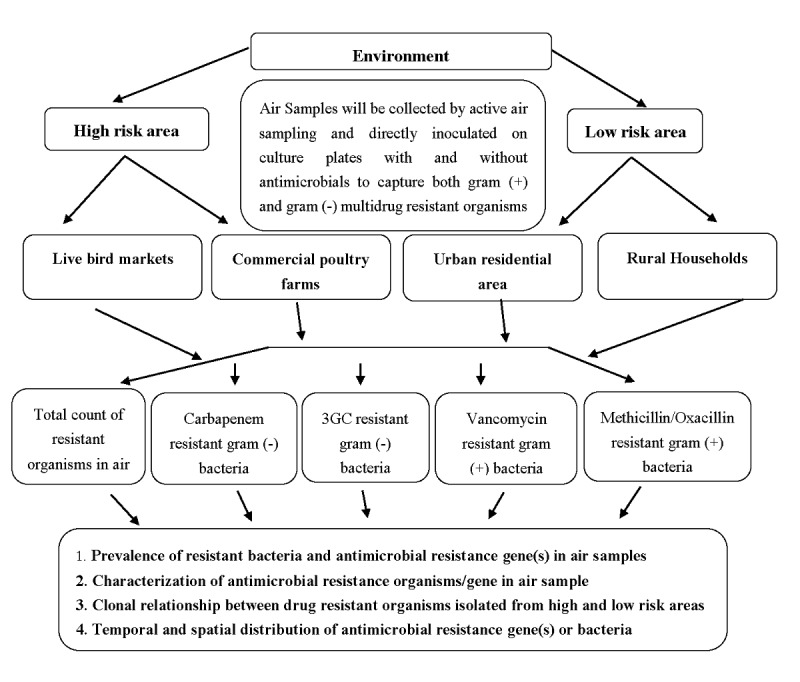
Sampling strategy and framework.

This is a cross-sectional pilot study designed to collect air samples from high-risk and low-risk environments in urban and periurban settings of Bangladesh, mainly the Dhaka metropolitan area and Mirzapur Upazilla (subdistrict) of Tangail District. Environmental systematic and grid sampling will be followed according to approaches described by Keith [35]. This is suitable for finding hotspots for target organisms/genes over a specific period of time, as samples are taken at regularly spaced intervals. Data collection started in January 2018, and the laboratory analysis is currently ongoing. The live bird markets and commercial poultry farms are considered to be high-risk areas, while the urban residential areas and the periurban households are low-risk areas (Figure 1).

### Geospatial Mapping

Mapping with GIS software (ArcGIS, Esri, Redlands, California) will plot concentrations of resistant genes and antimicrobial-resistant bacteria in environmental compartments (high- and low-risk areas) in each location using GPS coordinates. Temporal variation will be observed by comparing the magnitude of resistant bacteria and genes in the dry season with those in the wet season.

### Collection of Air Samples From Study Sites

**Figure 2:**
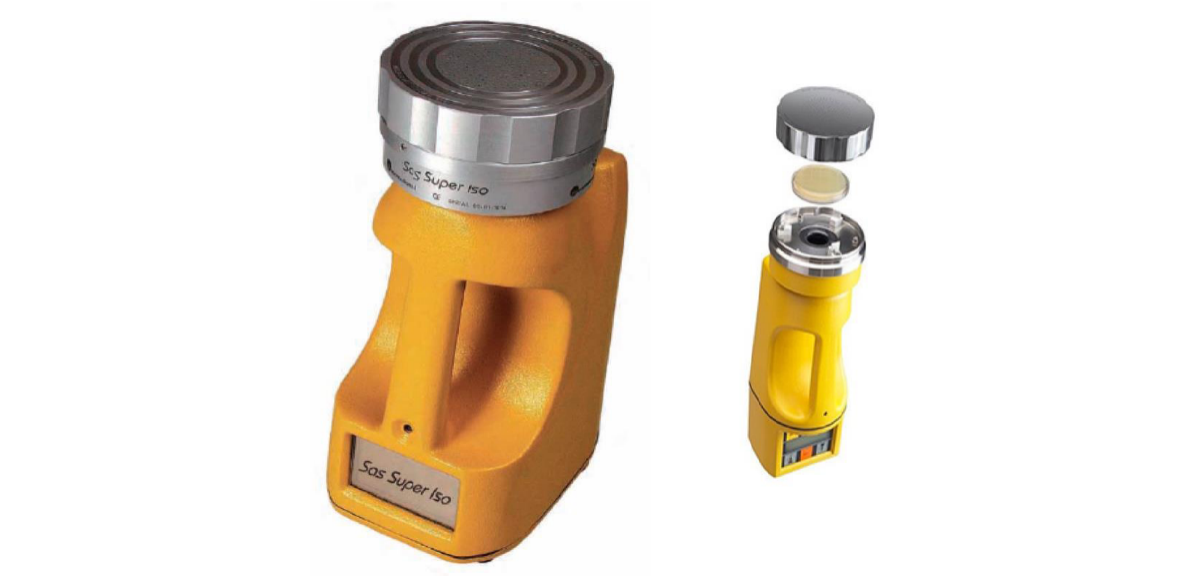
Active microbial air sampler.

Air samples from commercial poultry farms, live bird markets, rural households, and urban residential areas will be collected. All active sampling will be performed using the same Surface Air System Sampler (SAS Super 180 Microbial Air Sampler, Bioscience International, Rockville; Figure 2), with a flow rate of 180 L/min. The following media will be used for collection of samples: standard plate count (SPC) agar (Oxoid, Hampshire, United Kingdom) for aerobic plate count, MacConkey agar (BD Difco, Becton Dickinson, New Jersey) supplemented with cefotaxime (1 mg/L) and MacConkey agar supplemented with meropenem (0.5 mg/L) to obtain gram-negative resistant organisms, Mannitol Salt agar (MSA) (Oxoid) supplemented with oxacillin (8 mg/L), and Slanetz and Bartley (SB) agar (Oxoid) medium supplemented with vancomycin (6 mg/L) to obtain gram-positive resistant organisms. During sampling, the aspirating head will be removed from the air sampler. An identified, closed, and prepared plate will be inserted, and the plate lid will be removed. The aspirating head will be replaced again to cover the plate. The required volume and air flow rate will be adjusted, and the air sampler will be started. Subsequently, the airflow will be directed into the agar surface of the plate and at the end of a cycle, the aspirating head will be removed. The plate will be closed with the lid and removed from the air sampler. An identical method will be followed for different types of agar plates in each location, although the air volume will be different (ie, 1000 L for MacConkey, MSA, and SB agar and 100 L for SPC agar). After sampling, the plates will be carried in a carrying case and transported to the laboratory.

### Processing and Laboratory Analysis of Air Samples

#### Analysis of Antibiotic Resistance Genes in the Air Metagenome

According to the manufacturers’ instruction and using QIAamp DNA Mini Kit (QIAGEN, Germany), total DNA from the culture sweeps will be extracted after counting colonies on SPC agar plates. The colonies will then be incubated for 44 hours at 37°C. High-throughput sequencing will be performed using an Illumina MiSeq sequencing system (Illumina, San Diego, CA). The downstream analysis quality will be ensured by removing raw reads, with an average quality score below 20 or with length less than 100 bp (101 bp in length) or having three or more ambiguous nucleotides. The high-throughput sequencing will be carried out by the “Index 101 PE” (paired-end sequencing; 101-bp reads and 8-bp index sequence) sequencing strategy. There will be almost the same quantity of clean reads in this manner for each sample. The processing of the call sequences as well as raw fluorescent images will be carried out through a base-calling pipeline (Sequencing Control Software, Illumina). Metagenomic analyses will be carried out with the filtered clean reads (almost 1.6 GB per sample) after removal of the raw reads contaminated by an adapter (>15 bp overlap) or having three or more “N” [36,37]. The antibiotic resistance genes in the samples will be identified through alignment of the Illumina sequencing reads via offline BLAST (Basic Local Alignment Search Tool) against a self-established database. Similarity above 90% and alignments ≥25 amino acids are the identification criteria of a read to be confirmed as an antimicrobial resistance gene based on its best BLAST hit (blastx) [36].

#### Culture of Samples to Identify Antibiotic-Resistant Gram-Positive and Gram-Negative Organisms

All the culture plates (except SPC) exposed to air during air sampling will be incubated at 37°C, with 44 hours for SB agar and 18-24 hours for MacConkey agar and MSA. After incubation, each plate will be counted for both types of colonies (typical and atypical). To achieve clean/pure cultures, 3-4 phenotypically different isolated colonies will be subcultured from each plate on corresponding antibiotic-supplemented agar media. Following incubation (mentioned earlier), culture sweeps from each plate will be dissolved in tryptic soy broth cryovials containing 30% glycerol. These will be preserved at –80°C for future usage.

#### Antimicrobial Susceptibility Testing

By using different antibiotic discs, the agar diffusion test will determine the antimicrobial susceptibility as per the Clinical and Laboratory Standards Institute (CLSI 2016) guidelines [38]. Third-generation cephalosporin-resistant isolates will be tested for extended spectrum beta-lactamase (ESBL) production by performing combination disc tests. Vancomycin-resistant *Enterococci* (VRE), carbapenem-resistant *Enterobacteriaceae* (CRE), and methicillin-resistant *Staphylococcus aureus* (MRSA) will be confirmed by reviewing the susceptibility test results.

#### Detection of Antibiotic Resistance Genes

Polymerase chain reaction for genes specific for each of the resistance phenotypes (for ESBL: CTX-M, TEM, SHV; for carbapenem: NDM; for VRE: vanA; for MRSA: mecA) will be carried out according to the procedures described previously [39-42].

#### Genetic Fingerprinting of Isolates

Phenotypically same isolates (with or without similar resistance pattern) obtained from different locations or time periods will be further tested by pulsed-filed gel electrophoresis to determine their clonal diversity and dispersion.

#### Test for Antibiotic Resistance Plasmid

Plasmid DNA extraction and analysis from resistant isolates will be executed through the rapid alkaline lysis method and horizontal gel electrophoresis in 0.8% agarose gels, respectively [43]. The unknown plasmid size will be assumed by using known standard plasmid marker following gel electrophoresis for which the plasmids *Escherichia coli* V517 (1.4, 1.8, 2.0, 2.6, 3.4, 3.7, 4.8 and 35.8 MDa), pDK9 (140 MDa), R1 (62 MDa), RP4 (36 MDa), and Sa (23 MDa) will be considered as standards. Both filter mating and broth mating assays will be performed for conjugation at 30°C for 18 hours. The donor will be the resistant bacterial isolates when *E. coli* J53 (AziR, F−) and *E. coli* MC1061 (SmR, F−, nonlactose fermenting) will be recipients. MacConkey agar will be used for the selection of both *E. coli* J53 and *E. coli* MC1061 transconjugants. However, MacConkey agar will contain sodium azide (100 mg/L) and cefotaxime (20 mg/L)/cefoxitin (16 mg/L) for *E. coli* J53 and ampicillin (50 mg/L) for *E. coli* MC1061 transconjugants. The antibiotic susceptibility test will be performed to confirm transconjugant colonies. Using the alkaline lysis method described above, plasmid DNA will be extracted from transconjugants [43].

#### Estimation of Sample Size

The primary objective of this study is to determine the prevalence of antibiotic resistant organisms with resistance gene characterization in air samples from different locations and during different seasons. However, there are no baseline data available about measuring antimicrobial resistance in air samples. No prior assumptions were possible to show the anticipated variation in different exposures or seasonal contexts, although some prior knowledge has been utilized [29]. In this exploratory study, we have estimated 5% probability and 95% confidence, which requires a sample size of 59, as per the formula for sample size calculation of pilot studies published by Rik Crutzen et al [44]. Owing to the large number of study settings, we have included 80 sampling units for each season, which includes 20 live bird markets, 20 rural poultry farms, 20 periurban households, and 20 urban residential areas. The sampling bias is expected to be reduced through this strategy and will provide robust findings based on repeating the sampling in wet and dry season conditions.

### Outcome Variables

The outcome variables that will be assessed are as follows:

1. The prevalence of antibiotic-resistant organisms and resistance genes (positive occurrence of resistant bacteria/genes as a proportion of the number of samples) from each environmental location
2. The geospatial distribution of antibiotic resistant organisms and antibiotic resistance genes in study areas
3. The temporal prevalence of resistant bacteria and concentration of resistant genes in dry and wet season to assess seasonal variation in antimicrobial resistance
4. The identification of high-risk environments for air borne pollution with antimicrobial resistance

#### Data Analysis Plan

In this study, we will determine the presence of resistant organisms in air samples by total counting (colony-forming units per liter) at different study sites in Bangladesh. Significant differences in carriage rates of antibiotic-resistant organisms (and concentrations of resistant genes) in high- and low-risk environments will be determined by Chi-square and independent *t* tests. Hence, significant predictors will be identified. For identification of significant risk factors, logistic regression analysis will be used. Repeated measures analyses will be applied to examine whether there is significant seasonal variation in the prevalence of antimicrobial resistant bacteria and concentrations of antimicrobial resistance genes (paired *t* test or repeated measures analysis of variance). Count regression models like Poisson, negative binomial, and zero inflated count will be used, where appropriate, for antibiotic resistance count data.

## Results

The Research Review Committee and Ethical Review Committee of International Centre for Diarrhoeal Disease Research, Bangladesh, have approved this research protocol (protocol number: PR-17048). A unique study identification number was assigned to all air samples to ensure anonymity of the study sites. The study started in January 2018, and the collection of air samples was completed in November 2018. We have received 800 air samples from 80 study locations in both dry and wet seasons. Currently, the laboratory analysis is ongoing, and we expect to receive the microbiological results by October 2019. After completion of data cleaning and analysis, the manuscript submission is anticipated to be submitted before fall 2020. In addition to publication in a high-impact, peer-reviewed journal and as per the dissemination plan, the study results will be shared with the study participants, with scientific communities, with relevant government authorities, and in related conferences or workshops.

## Discussion

### Overview

The rise and spread of superbugs have become a key public health and planetary health concern worldwide. Infections caused by multidrug-resistant bacteria are linked to greater mortality rates than antimicrobial-susceptible bacteria [45]. For therapeutic purposes, medically important antimicrobials are used extensively in agricultural and farming industries for disease prevention (eg, prophylaxis and metaphylaxis), treatments, and growth promotions. More than two-thirds of antimicrobials are consumed in the livestock sector each year. It is projected that by 2030, there may be a massive increase (up to 67%) in global antimicrobial use in food-producing animals, especially in Brazil, Russia, India, China, and South Africa (BRICS) [46]. The antimicrobial resistance problem is severe, not only in developed countries but also in nonindustrialized countries. Scarcity of antimicrobial usage policies and substandard hygiene situations are the precipitating factors that drive antimicrobial resistance issue to be more challenging [47].

The burden of the antimicrobial resistance rate depends on the population of a country and its environment. Bangladesh is a highly populated country; therefore, the rate of antimicrobial resistance is extensive due to rapid spread of antimicrobial-resistant organisms. Antimicrobial resistance genes are conferring their resistance value to a wider community including both animals and humans, through close interactions with the environment and the wastes that are disposed in the environment, directly affecting the food chain [48]. Due to the extensive use of antibiotics as therapeutic and prophylactic in farming, bacterial resistance may be developed by either chromosomal gene mutation or gene acquisition through different mechanisms like transformation, transduction, or conjugation [49]. Continuous accumulation of multiple mutations have caused the genome to become resistant to antibiotics. To screen the antimicrobial resistance genes, whole genome sequencing is an important tool for understanding the antimicrobial resistance mechanisms. Whole genome sequencing helps evaluate the number of mutations and particular mechanisms of the mutated gene that drive antimicrobial resistance to develop new drugs (antibiotic) and diagnose the disease state and treatment process [47]. Primary biological aerosol particles such as bacteria, viruses, pollens, and mold spores are important components of airborne particulate matter (PM), and abundant pathogenic bacteria have been identified from PM2.5 and PM10 samples [50]. However, these compounds from pollution and other sources are not prioritized during analysis. Our study will also focus on the resistant bacteria as an integral part of air pollution.

Intrinsic genetic determinants of resistance factors are harbored by bacteria with macromolecules in the environment. Robust evidence suggests that such macromolecules developing “environmental resistomes” are a source from which clinically relevant bacteria acquire antibiotic resistance genes [51]. Poultry, the most common rural domestic species, is considered a major driver for selection of antimicrobial resistance from the environment to human/animal due to fecal shedding of resistant bacteria. As poultry feces are used as fertilizers and household members share sleeping spaces with poultry animals, antibiotic use in poultry in the last 6 months has been reported by more than 50% poultry owners [37]. Commercial poultry farm areas harbor the antimicrobial-resistant isolates in the air, and humans are easily exposed to these antimicrobial-resistant isolates during inhalation of dust and particles. Consequently, antimicrobial-resistant organisms can build up a strong biofilm in the intestinal tract of humans. Through excretion of stool and other body fluids, these can be spread in the environment. Urban live bird markets are the largest site of bird slaughtering, with no proper waste management setting, and the huge amounts of wastes are directly passed into environment through dust and direct wash out [14].

The importance of environmental compartments as the transmission hub of antimicrobial resistance is well established. However, airborne resistomes and their transmission pathway are poorly studied. Without containment of environmental reservoirs, antimicrobial resistance prevention policy will fail [52]. The occurrence of pathogenic resistant organisms and resistance genes in atmospheric air of different locations will guide researchers and policy makers to adopt new strategies for the containment of this alarming issue. However, further large-scale studies need to be carried out to link the clinically important organisms and airborne resistomes. To achieve this, One Health surveillance needs to be carried out in humans (both healthy and clinical patients), animals, and environments at the national level.

### Strengths and Limitations

Regarding the strengths, our study will focus on the air resistomes, which is a less explored environmental dimension of antimicrobial resistance transmission dynamics. To our knowledge, this is the first study to explore the presence of superbugs in the air in commonly exposed areas in Bangladesh. The limitation exists in the small sample size and lack of baseline data from the atmospheric environment of Bangladesh. Therefore, the findings may not be generalizable for all areas in the country.

## Appendix

Multimedia Appendix 1 Peer-reviewer report from the International Centre for Diarrhoeal Disease Research, Bangladesh.

## Abbreviations

BLAST: Basic Local Alignment Search Tool
CRE: Carbapenem-resistant *Enterobacteriaceae*
CLSI: Clinical and Laboratory Standards Institute
ESBL: Extended-Spectrum Beta-Lactamase
MRSA: Methicillin-resistant *Staphylococcus aureus*
VRE: Vancomycin-resistant *Enterococci*

## References

[1] O’Neill J (2014). Review on Antimicrobial Resistance:Tackling drug-resistant infections globally.

[2] Martinez JL (2009). Environmental pollution by antibiotics and by antibiotic resistance determinants. Environ Pollut.

[3] Baquero F, Martínez José-Luis, Cantón Rafael (2008). Antibiotics and antibiotic resistance in water environments. Curr Opin Biotechnol.

[4] van Hoek AHAM, Mevius D, Guerra B, Mullany P, Roberts AP, Aarts HJM (2011). Acquired antibiotic resistance genes: an overview. Front Microbiol.

[5] D'Costa VM, McGrann KM, Hughes DW, Wright GD (2006). Sampling the antibiotic resistome. Science.

[6] Kim S, Carlson K (2007). Temporal and Spatial Trends in the Occurrence of Human and Veterinary Antibiotics in Aqueous and River Sediment Matrices. Environ Sci Technol.

[7] Yang J, Ying G, Zhao J, Tao R, Su H, Liu Y (2011). Spatial and seasonal distribution of selected antibiotics in surface waters of the Pearl Rivers, China. J Environ Sci Health B.

[8] Ham Y, Kobori H, Kang J, Matsuzaki T, Iino M, Nomura H (2012). Distribution of antibiotic resistance in urban watershed in Japan. Environ Pollut.

[9] Knapp CW, Lima L, Olivares-Rieumont S, Bowen E, Werner D, Graham DW (2012). Seasonal variations in antibiotic resistance gene transport in the almendares river, havana, cuba. Front Microbiol.

[10] Gandolfi I, Bertolini V, Ambrosini R, Bestetti G, Franzetti A (2013). Unravelling the bacterial diversity in the atmosphere. Appl Microbiol Biotechnol.

[11] Zhao S, Liu X, Cheng D, Liu G, Liang B, Cui B, Bai J (2016). Temporal-spatial variation and partitioning prediction of antibiotics in surface water and sediments from the intertidal zones of the Yellow River Delta, China. Sci Total Environ.

[12] Liu F, Xu X, Tu B, Wang C, Jiang X, Wang L, Xue Y (2019). Distribution Characteristics of Antibiotic Resistance Genes in PM of a Concentrated Broiler Feeding Operation [in Chinese]. Huan Jing Ke Xue.

[13] Li Y, Liao H, Yao H (2019). Prevalence of Antibiotic Resistance Genes in Air-Conditioning Systems in Hospitals, Farms, and Residences. Int J Environ Res Public Health.

[14] Zhang M, Zuo J, Yu X, Shi X, Chen L, Li Z (2018). Quantification of multi-antibiotic resistant opportunistic pathogenic bacteria in bioaerosols in and around a pharmaceutical wastewater treatment plant. J Environ Sci (China).

[15] Shi X, Wang S (2018). Antibiotic resistance in environment of animal farms [in Chinese]. Sheng Wu Gong Cheng Xue Bao.

[16] Rosen K, Roesler U, Merle R, Friese A (2018). Persistent and Transient Airborne MRSA Colonization of Piglets in a Newly Established Animal Model. Front Microbiol.

[17] Matinyi S, Enoch M, Akia D, Byaruhanga V, Masereka E, Ekeu I, Atuheire C (2018). Contamination of microbial pathogens and their antimicrobial pattern in operating theatres of peri-urban eastern Uganda: a cross-sectional study. BMC Infect Dis.

[18] Jiang Meijie, Mu Yunqing, Li Ning, Zhang Zhijun, Han Shulin (2018). Carbapenem-resistant from Air and Patients of Intensive Care Units. Pol J Microbiol.

[19] Gao X, Shao M, Wang Q, Wang L, Fang W, Ouyang F, Li J (2018). Airborne microbial communities in the atmospheric environment of urban hospitals in China. J Hazard Mater.

[20] Brągoszewska E, Biedroń I (2018). Indoor Air Quality and Potential Health Risk Impacts of Exposure to Antibiotic Resistant Bacteria in an Office Rooms in Southern Poland. Int J Environ Res Public Health.

[21] Solomon FB, Wadilo F, Tufa EG, Mitiku M (2017). Extended spectrum and metalo beta-lactamase producing airborne Pseudomonas aeruginosa and Acinetobacter baumanii in restricted settings of a referral hospital: a neglected condition. Antimicrob Resist Infect Control.

[22] Gao M, Jia R, Qiu T, Han M, Wang X (2017). Size-related bacterial diversity and tetracycline resistance gene abundance in the air of concentrated poultry feeding operations. Environ Pollut.

[23] Gao X, Shao M, Luo Y, Dong Y, Ouyang F, Dong W, Li J (2016). Airborne bacterial contaminations in typical Chinese wet market with live poultry trade. Sci Total Environ.

[24] Blaak H, van Hoek AHAM, Hamidjaja RA, van der Plaats RQJ, Kerkhof-de Heer L, de Roda Husman AM, Schets FM (2015). Distribution, Numbers, and Diversity of ESBL-Producing E. coli in the Poultry Farm Environment. PLoS One.

[25] Liu D, Chai T, Xia X, Gao Y, Cai Y, Li X, Miao Z, Sun L, Hao H, Roesler U, Wang J (2012). Formation and transmission of *Staphylococcus aureus* (including MRSA) aerosols carrying antibiotic-resistant genes in a poultry farming environment. Sci Total Environ.

[26] Brooks J, McLaughlin M, Scheffler B, Miles D (2010). Microbial and antibiotic resistant constituents associated with biological aerosols and poultry litter within a commercial poultry house. Sci Total Environ.

[27] Sapkota A, Ojo K, Roberts M, Schwab K (2006). Antibiotic resistance genes in multidrug-resistant Enterococcus spp. and Streptococcus spp. recovered from the indoor air of a large-scale swine-feeding operation. Lett Appl Microbiol.

[28] Gaze WH, Krone SM, Larsson DJ, Li X, Robinson JA, Simonet P, Smalla K, Timinouni M, Topp E, Wellington EM, Wright GD, Zhu Y (2013). Influence of humans on evolution and mobilization of environmental antibiotic resistome. Emerg Infect Dis.

[29] Pehrsson EC, Tsukayama P, Patel S, Mejía-Bautista Melissa, Sosa-Soto G, Navarrete KM, Calderon M, Cabrera L, Hoyos-Arango W, Bertoli MT, Berg DE, Gilman RH, Dantas G (2016). Interconnected microbiomes and resistomes in low-income human habitats. Nature.

[30] Huijbers PMC, Blaak H, de Jong MCM, Graat EAM, Vandenbroucke-Grauls CMJE, de Roda Husman AM (2015). Role of the Environment in the Transmission of Antimicrobial Resistance to Humans: A Review. Environ Sci Technol.

[31] Forsberg KJ, Reyes A, Wang B, Selleck EM, Sommer MOA, Dantas G (2012). The shared antibiotic resistome of soil bacteria and human pathogens. Science.

[32] Humeniuk C, Arlet G, Gautier V, Grimont P, Labia R, Philippon A (2002). Beta-lactamases of Kluyvera ascorbata, probable progenitors of some plasmid-encoded CTX-M types. Antimicrob Agents Chemother.

[33] Poirel L, Rodriguez-Martinez J, Mammeri H, Liard A, Nordmann P (2005). Origin of Plasmid-Mediated Quinolone Resistance Determinant QnrA. Antimicrobial Agents and Chemotherapy.

[34] Lemmen S, Häfner H, Zolldann D, Stanzel S, Lütticken R (2004). Distribution of multi-resistant Gram-negative versus Gram-positive bacteria in the hospital inanimate environment. J Hosp Infect.

[35] Keith L (1991). Environmental Sampling and Analysis: A Practical Guide, 1st Edition.

[36] Huang K, Tang J, Zhang X, Xu K, Ren H (2014). A comprehensive insight into tetracycline resistant bacteria and antibiotic resistance genes in activated sludge using next-generation sequencing. Int J Mol Sci.

[37] Roess AA, Winch PJ, Akhter A, Afroz D, Ali NA, Shah R, Begum N, Seraji HR, El Arifeen S, Darmstadt GL, Baqui AH, Bangladesh Projahnmo Study Group (2015). Household Animal and Human Medicine Use and Animal Husbandry Practices in Rural Bangladesh: Risk Factors for Emerging Zoonotic Disease and Antibiotic Resistance. Zoonoses Public Health.

[38] (2016). Clinical and Laboratory Standards Institute.

[39] Talukdar PK, Rahman M, Rahman M, Nabi A, Islam Z, Hoque MM, Endtz HP, Islam MA (2013). Antimicrobial resistance, virulence factors and genetic diversity of Escherichia coli isolates from household water supply in Dhaka, Bangladesh. PLoS One.

[40] Islam MA, Talukdar PK, Hoque A, Huq M, Nabi A, Ahmed D, Talukder KA, Pietroni MAC, Hays JP, Cravioto A, Endtz HP (2012). Emergence of multidrug-resistant NDM-1-producing Gram-negative bacteria in Bangladesh. Eur J Clin Microbiol Infect Dis.

[41] Kim M, Cha M, Ryu J, Woo G (2017). Characterization of Vancomycin-Resistant Enterococcus faecalis and Enterococcus faecium Isolated from Fresh Produces and Human Fecal Samples. Foodborne Pathog Dis.

[42] Sharma NK, Rees CED, Dodd CER (2000). Development of a single-reaction multiplex PCR toxin typing assay for *Staphylococcus aureus* strains. Appl Environ Microbiol.

[43] Kado CI, Liu ST (1981). Rapid procedure for detection and isolation of large and small plasmids. J Bacteriol.

[44] Viechtbauer W, Smits L, Kotz D, Budé Luc, Spigt M, Serroyen J, Crutzen R (2015). A simple formula for the calculation of sample size in pilot studies. J Clin Epidemiol.

[45] Araos R, Tai AK, Snyder GM, Blaser MJ, D'Agata EMC (2016). Predominance of Lactobacillus spp. Among Patients Who Do Not Acquire Multidrug-Resistant Organisms. Clin Infect Dis.

[46] Brower CH, Mandal S, Hayer S, Sran M, Zehra A, Patel SJ, Kaur R, Chatterjee L, Mishra S, Das B, Singh P, Singh R, Gill J, Laxminarayan R (2017). The Prevalence of Extended-Spectrum Beta-Lactamase-Producing Multidrug-Resistant Escherichia Coli in Poultry Chickens and Variation According to Farming Practices in Punjab, India. Environ Health Perspect.

[47] Abdelgader SA, Shi D, Chen M, Zhang L, Hejair HMA, Muhammad U, Yao H, Zhang W (2018). Antibiotics Resistance Genes Screening and Comparative Genomics Analysis of Commensal Isolated from Poultry Farms between China and Sudan. Biomed Res Int.

[48] Holvoet K, Sampers I, Callens B, Dewulf J, Uyttendaele M (2013). Moderate Prevalence of Antimicrobial Resistance in Escherichia coli Isolates from Lettuce, Irrigation Water, and Soil. Appl Environ Microbiol.

[49] da Costa P, Loureiro L, Matos A (2013). Transfer of multidrug-resistant bacteria between intermingled ecological niches: the interface between humans, animals and the environment. Int J Environ Res Public Health.

[50] Liu H, Zhang X, Zhang H, Yao X, Zhou M, Wang J, He Z, Zhang H, Lou L, Mao W, Zheng P, Hu B (2018). Effect of air pollution on the total bacteria and pathogenic bacteria in different sizes of particulate matter. Environ Pollut.

[51] Munita JM, Arias CA (2016). Mechanisms of Antibiotic Resistance. Microbiol Spectr.

[52] Asaduzzaman M (2018). Antimicrobial resistance: an urgent need for a planetary and ecosystem approach. Lancet Planet Health.

