# Supplementary material for: Quantification of Airborne Resistant Organisms With Temporal and Spatial Diversity in Bangladesh: Protocol for a Cross-Sectional Study
Source: JMIR Res Protoc. 2019 Dec 19;8(12):e14574. doi: 10.2196/14574 (PMC6940864; doi:10.2196/14574)
Supplement: Multimedia Appendix 1 [file resprot_v8i12e14574_app1.pdf]

icddr,b

Memorandum

23 April 2017

To: Dr Muhammad Asaduzzaman
Principal Investigator of research protocol # PR-17048
Laboratory Sciences and Services Division (LSSD)

From: Shafiqul Alam Sarker, MD, PhD SASarker
Chairperson
Research Review Committee (RRC)

Sub: Research protocol # PR-17048

Thank you for submitting your research protocol # PR-17048 titled "Occurrence of multidrug resistant organisms and antimicrobial resistance genes in the outdoor environment of Bangladesh with their temporospatial diversity" for consideration of the RRC and present it before the Committee in its special meeting held on 13 April 2017. This is to inform you that after review and discussion, the committee made the following observations on the protocol:

a) In the protocol live poultry market of Dhaka Metropolitan area and commercial poultry farm of Mirzapur Upazilla are included as high risk environment and residential area of Dhaka metropolitan a households of Mirzapur Upazilla as low risk environment. These areas do not represent the entire outdoor environment of Bangladesh. So the title of the protocol should be rephrased accordingly.

b) In this protocol only the poultry associated environmental risk has been included as high risk environment - a justification should be provided in favour of this statement

c) In page-3, sex of the Co-Principal Investigator has not been checked out.

d) In page-5, as is no consent process will be required in the protocol, question of checking language of the consent does not arise. It should be mentioned not applicable.

e) In page-6, in the chapter of Biological specimen, in item (b) tests for the preserved specimens should be mentioned.

f) In the project summary, in page-11, in the chapter of Knowledge gap in(b) the language seemed confusing. It appeared that resistant organisms and antibiotic resistant genes exist separately in air. In fact, it should be antibiotic resistant bacteria and the genes conferring resistance to antibiotics carried by these bacteria, as has been stated in the hypothesis (d) in page-11. The language of the knowledge gap Chapter should be changed accordingly. The language of Objective-1 and Objective-4 should also changed in line of the language of the hypothesis.

GPO Box 128, Dhaka 1000, Bangladesh.
For Packages : 68, Shaheed Tajuddin Ahmed Sarani, Mohakhali, Dhaka 1212, Bangladesh.
Phone : 880-2-9827001-10, Web : http://www.icddrb.org, Fax : 880-2-9827077

g) In the description of Research Project (page-12) 4 objectives mentioned in the project summery (page 11) have been listed as "Specific objectives". This should be sorted out.

h) Justification should be given for selecting Dhaka metropolitan area and Mirzapur Upazilla as study sites.

i) Brief description of "Active microbial sampler (figure-1, page-14) should be provided.

j) Last lines in page-17, samples from Round-1 and Round-2 are not clearly understood.

You are, therefore, advised to address each of the above mentioned observations of the committee and submit the revised version of the protocol for consideration by the chair.

Thank you once again.

Cc: Senior Director, LSSD
